# From Birth to Midlife—Liver Function, Fibrosis and Mortality in Individuals with Severe Alpha-1-Antitrypsin Deficiency Identified by Neonatal Screening

**DOI:** 10.3390/jcm15072553

**Published:** 2026-03-27

**Authors:** Georg Rüdiger Schramm, Mohammed Abdulrasak, Suneela Zaigham, Eeva Piitulainen, Hanan Tanash

**Affiliations:** 1 Department of Respiratory Medicine and Allergology, Lund University, Skåne University Hospital, Tora Kjellgrens Gata 17, 20502 Malmö, Sweden; suneela.zaigham@med.lu.se (S.Z.); eeva.piitulainen@med.lu.se (E.P.); hanan.tanash@med.lu.se (H.T.); 2 Department of Clinical Sciences, Lund University, 22100 Malmö, Sweden; mohammed.abdulrasak@med.lu.se; 3 Department of Gastroenterology and Nutrition, Skåne University Hospital, 21428 Malmö, Sweden

**Keywords:** Alpha-1-Antitrypsin deficiency, liver disease

## Abstract

**Background**: Severe Alpha-1-Antitrypsin deficiency (AATD), phenotype PiZZ, is a leading cause of liver disease in neonates, children, and adults. Nevertheless, the prevalence of liver disease and mortality within PiZZ adults remains unclear. Between 1972 and 1974, a cohort of 129 individuals with severe AATD (PiZZ) was identified through the Swedish national screening of 200,000 newborns. The cohort has been followed up regularly since birth. This prospective cohort follow-up study, with a cross-sectional comparison at 50 years of age, aims to characterize the natural history of liver disease and mortality in this cohort in their early fifties, compared with an age-matched control group (PiMM) randomly selected from the population registry. **Methods**: Study participants completed questionnaires regarding occupation, medical history, medication, and alcohol consumption. They underwent physical examination and measurement of liver stiffness using transient elastography (TE, FibroScan^®^). Blood samples were obtained for evaluation of liver function, alcohol consumption, calculation of liver fibrosis scores, and detection of viral hepatitis and autoimmune liver disease. **Results**: Ninety-five PiZZ and 124 PiMM individuals participated in the study, of whom 47 PiZZ and 96 PiMM underwent TE measurement. PiZZ individuals had significantly higher median liver stiffness compared with PiMM individuals (5.9 kPa vs. 4.5 kPa, *p* < 0.01). No significant differences were found in Fib-4 score or the Non-Alcoholic Fatty Liver Disease Fibrosis Score (NFS) between the groups. Since identification of the cohort at birth, 13 (10%) of the 129 PiZZ individuals have died. Of these, liver disease was the main or underlying cause of death in 8 individuals (6%). **Conclusions**: In their early fifties, PiZZ individuals show a small but significant increase in liver stiffness measured by TE, indicating early liver fibrosis. In contrast, conventional fibrosis scores, such as Fib-4 and NFS, do not differ between PiZZ individuals and PiMM, suggesting that serum-based fibrosis scores may underestimate fibrosis in AATD. In this cohort, liver disease and its complications represented the main cause of death in PiZZ individuals by the age of 50, an observation that is uncommon in the general population at this age.

## 1. Introduction

Alpha-1-Antitrypsin deficiency (AATD) is an autosomal codominant disorder caused by mutations in the SERPINA1 gene on chromosome 14. Alpha-1-Antitrypsin (AAT) is a 52-kDa protein, primarily synthesized in the hepatocytes within the endoplasmic reticulum (ER) and secreted via the Golgi apparatus into the circulation. In individuals with severe AATD, phenotype PiZZ, most AAT is degraded in the ER. The defective Z protein (Glu342Lys substitution) leads to accumulation of misfolded AAT in the ER, resulting in decreased AAT serum levels, ER stress and a mitochondrial dysfunction [1,2].

Emphysema is the most prevalent clinical disorder associated with severe AAT deficiency and represents the leading cause of morbidity and mortality in affected individuals [3,4].

Liver disease is the second most common clinical manifestation and typically presents in infancy with cholestasis. Severity ranges from transient neonatal cholestasis—observed in approximately 15% of affected newborns—to progressive liver fibrosis and cirrhosis [5]. In adulthood, severe AAT deficiency may manifest as asymptomatic elevations in liver enzymes, established cirrhosis, or hepatocellular carcinoma (HCC) [6,7]. Less commonly, affected individuals may also develop panniculitis or vasculitis.

Since routine tests, such as liver function tests (LFTs) and ultrasound scans (USS), may lack sensitivity in detecting early and reversible liver impairment, liver involvement may remain undetected in the absence of systematic and comprehensive evaluation [8].

Although liver biopsy has historically been considered the gold standard for diagnosing liver disease, its associated morbidity and mortality limit its suitability for routine clinical use [9]. Consequently, a range of non-invasive diagnostic approaches has been developed, including liver stiffness measurement by elastography and several serum-based fibrosis scoring systems, such as the FibroTest, FibroMeter, AST-to-platelet ratio index (APRI), enhanced liver fibrosis (ELF) test, Fib-4, and the NAFLD fibrosis score (NFS). These methods are increasingly replacing liver biopsy for both initial assessment and longitudinal monitoring [10]. However, most non-invasive fibrosis scores were developed and validated in populations with viral hepatitis or metabolic liver disease [11,12]. Their applicability in genetic liver diseases, such as AATD, remains insufficiently studied, highlighting the need for further evaluation in these populations.

Between 1972 and 1974, 200,000 newborns in Sweden were screened for AATD to study its natural history. This screening identified a cohort of AAT-deficient individuals, including 127 PiZZ, 2 PiZnull, 54 PiSZ, and 1 PiSnull. The cohort has been followed regularly since birth. Follow-up assessments of this cohort during adulthood have not shown signs of liver disease among PiZZ individuals before the age of 38 years [6,13,14].

Despite decades of follow-up, the prevalence of liver disease and the fibrosis burden in middle-aged PiZZ adults remain insufficiently characterized. Earlier studies indicate that development of liver disease in AAT deficiency appears stable before the age of 40, with increased evidence of fibrosis progression thereafter [15].

This prospective study aimed to investigate early signs of liver disease in 50-year-old individuals with severe AATD (PiZZ) compared with an age-matched control group, and to identify non-invasive methods for detecting liver fibrosis as well as risk factors associated with liver disease. In addition, liver-related morbidity and mortality, along with overall mortality, were assessed within the cohort.

## 2. Materials and Methods

### 2.1. The Study Population

All PiZZ individuals in the cohort were invited to participate in the study. The same 300 age-matched control subjects, who had been randomly selected from the Swedish population registry at the age of 30 years and had served as controls at the 34-, 38- and 42-year follow-ups, were invited to participate as the control group (PiMM). However, participation rates differed across earlier follow-ups, and the participants therefore may not fully overlap between follow-up visits, which could influence comparability at age 50. The check-up of the cohort was conducted between February 2020 and November 2023.

Questionnaires and informed consent forms were sent to PiZZ individuals and controls. After providing written consent and completing the questionnaires, participants were invited to the study site for further clinical investigations, including measurement of transient elastography (TE) and spirometry, at the Department of Respiratory Medicine, Skåne University Hospital (DRM-SUS), Malmö, Sweden. Since PiZZ individuals reside throughout Sweden, some were unable to attend the study site and preferred to provide blood samples and undergo spirometry at their local hospital. However, TE and full clinical examinations could not be performed at local hospitals. Participants who did not respond to the questionnaires were contacted by phone when possible. For individuals without available contact information, we attempted to reach their next of kin. For participants who died during the study, we contacted their clinicians to determine the cause of death. Causes of death were obtained from local hospitals via death certificates. Therefore, at both the start and end of the study, we had information on the vital status of all PiZZ individuals identified during screening. Participants in the control group visited DRM-SUS in Malmö, Sweden.

### 2.2. Questionnaires

The questionnaire included questions about occupation, smoking habits, physical activity, comorbid conditions, prescription and over-the-counter medication, contraceptive use, and nutritional supplements. All medication, contraceptives and nutritional supplements regularly taken by the participants were assessed for potential liver strain using information available in the Swedish Pharmaceutical Specialties database (www.fass.se).

To assess alcohol consumption, study participants completed the Alcohol Use Disorders Identification Test (AUDIT), where scores of 0–7 indicate low risk, 8–15 indicate increasing risk, 16–19 indicate higher risk, and 20 or above suggest possible dependence [16,17].

All participants were asked if they had undergone a liver USS within last two years. USS was performed or recommended in all individuals with AATD, and in controls if they had abnormal blood tests or abnormal findings on TE. In Sweden, regular USS screening is recommended for individuals with AATD.

### 2.3. Physical Examinations

Physical examinations were performed at the DRM-SUS, Malmö, Sweden. The following anthropometric data were measured and recorded according to the study protocol: weight, height, Body Mass Index (BMI), blood pressure, and waist circumference (in cm, measured at a level midway between the lowest rib and the iliac crest). Obesity was defined as BMI ≥ 30 kg/m^2^. Metabolic syndrome was defined by the International Diabetes Federation criteria as central obesity (waist circumference ≥ 94 cm in men and ≥80 cm in women) plus at least two of the following: (1) triglycerides ≥ 150 mg/dL or specific treatment for hypertriglyceridemia; (2) high-density lipoprotein cholesterol < 40 mg/dL in men or <50 mg/dL in women or specific treatment for dyslipidemia; (3) systolic blood pressure ≥ 130 mmHg or diastolic blood pressure ≥ 85 mmHg or treatment for arterial hypertension; or (4) fasting glucose ≥ 100 mg/dL or previously diagnosed type 2 diabetes [18,19].

### 2.4. Liver Stiffness Measurement

Liver stiffness was measured using TE with the Echosens FibroScan^®^ 430 (Echosens, Paris, France). Participants fasted for at least 3 h and were positioned lying on their back with their right arm at maximum abduction. The probe was placed in the right mid-clavicular line between the 9th and 11th intercostal space. TE results were considered valid after 12 successful measurements with a range of ≤30%. Results are presented in kPa.

A TE value above 7.2 kPa was considered indicative of an increased risk of liver fibrosis [10].

### 2.5. Liver Stiffness Risk Scores

To allow comparison of serum-based fibrosis indices with TE results, both Fib-4 and NFS were calculated from the participants who underwent TE. Fib-4 was calculated by the formula: *age* (years) × *AST* (U/L) divided with [*Platelets* (×10^9^/L) × √*ALT* (U/L)]. The risk of liver fibrosis was considered low for scores below 1.3, intermediate for scores between 1.3 and 2.67, and high with a score of more than 2.67.

The NFS was calculated with the formula: −1.675 + 0.037 × *age* (years) + 0.094 × *BMI* (kg/m^2^) + 1.13 × *impaired glucose tolerance or diabetes mellitus* (*yes* = 1, *no* = 0) + 0.99 × *AST*/*ALT ratio* − 0.013 × *platelets* (×10^9^/L) − 0.66 × *albumin* (g/dL). The risk for liver fibrosis was considered low for scores below −1.455, intermediate for scores between −1.455 and 0.675, and high for scores above 0.675.

### 2.6. Laboratory Assessment

All laboratory samples were analyzed by the Department of Laboratory medicine, Skåne University Hospital, Malmö, Sweden. The following blood parameters were measured: aspartate transaminase (AST), alanine transaminase (ALT), alkaline phosphatase (ALP), gamma-glutamyltransferase (GGT), bilirubin, albumin, platelets, Alpha-1-Antitrypsin (AAT), phosphatidylethanols (PEth), hepatitis A IgM antibodies (anti-HAV IgM), hepatitis A antibodies (anti-HAV), hepatitis B surface antigen (HBsAg), hepatitis C antibodies (anti-HCV), antinuclear antibodies (ANA), smooth muscle antibodies (SMA), antimitochondrial antibodies (AMA), anti-neutrophil cytoplasmic antibodies (ANCA), proteinase 3 antibodies (PR3), myeloperoxidase antibodies (MPO), and glomerular basement membrane antibodies (GBM). Pathologically elevated values were defined as follows: AST > 36 U/L in women and >45 U/L in men, ALT > 45 U/L in women and >66 U/L in men, GGT > 72 U/L in women and >114 U/L in men, ALP > 114 U/L, bilirubin > 1500 U/L, albumin > 2700 U/L, and platelets < 165 × 10^9^/L in women and <145 × 10^9^/L in men. PEth was classified as “no or sporadic consumption” (<35 ng/mL), “moderate alcohol consumption” (35–200 ng/mL) and “heavy or regular consumption” (>200 ng/mL).

### 2.7. Statistical Analysis

Statistical analyses were performed using the Statistical Package for the Social Sciences (SPSS), version 28 (IBM Corp., Armonk, NY, USA). Continuous variables are presented as median values with ranges. The Mann–Whitney U test was used to compare continuous variables, while nominal variables were analyzed using Chi-squared tests. Binary logistic regression models were used to calculate the odds ratio (ORs) with 95% confidence intervals (95% CIs) for liver stiffness > 7.2 kPa, adjusted for AAT status, BMI, sex, and AUDIT score. A *p*-value below 0.05 was considered statistically significant.

## 3. Results

### 3.1. The Study Participants

**Figure 1:**
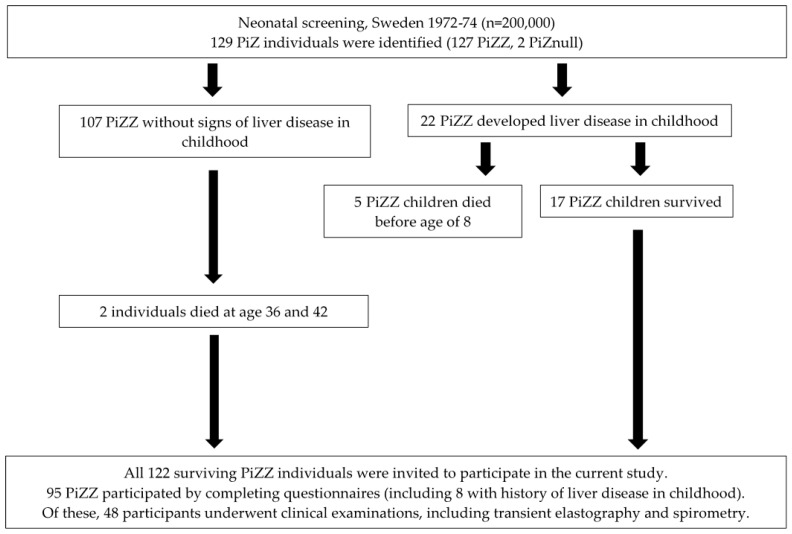
Flowchart showing the natural history of the cohort of PiZZ individuals from birth to the start of this study.

A total of 95 of the 122 PiZZ (78%) and 124 of the 300 PiMM individuals (42%) participated in the study (Figure 1, Table 1). One PiZZ subject had undergone liver transplantation and was excluded from the analyses.

The proportion of men was higher among the PiZZ individuals (*p* = 0.045), whereas the proportion of individuals with metabolic syndrome was higher within the PiMM individuals (*p* = 0.042).

Six percent of PiZZ individuals scored within the risk zone for alcohol overconsumption (AUDIT ≥ 8) and 38% had a PEth level above 35 ng/mL; however, neither differed significantly from PiMM. Compared with PiMM individuals, PiZZ participants had significantly higher median ALT (*p* = 0.007) and GGT (*p* = 0.005), as well as a higher proportion of pathologically elevated ALT (7% vs. 0%, *p* = 0.007, Appendix A, Table A1).

No significant differences were observed between the groups regarding the presence of autoimmune antibodies, hepatitis markers, or consumption of hepatotoxic medications.

### 3.2. Results of Transient Elastography

Forty-eight PiZZ individuals and 96 PiMM individuals underwent measurement of liver stiffness by TE (Table 2). Median liver stiffness was significantly higher among PiZZ individuals compared with PiMM (5.9 kPa vs. 4.9 kPa, *p* < 0.01). A significantly higher proportion of PiZZ individuals (26% vs. 9%, *p* = 0.01) had a liver stiffness value above 7.2 kPa compared with PiMM. No statistically significant differences were observed in the Fib-4 score or NFS between the groups. Among individuals who underwent TE, the PiZZ had a significantly higher ALT (*p* = 0.005) and GGT (*p* = 0.012), as well as a higher proportion of individuals with pathologically elevated ALT (*p* = 0.013), compared with PiMM (Appendix A, Table A3 and Table A4).

Cross-tabulation of transient elastography and Fib-4 classifications showed limited agreement between the methods. Among individuals with TE ≥ 7.2 kPa, 6 out of 11 (54.5%) had Fib-4 values below the fibrosis threshold, and 9 out of 10 had NFS values below the fibrosis risk threshold, indicating substantial discordance between transient elastography and the serum-based fibrosis score.

When stratified by BMI, liver stiffness was significantly higher in PiZZ individuals compared with PiMM individuals in both the BMI ≥ 30 and BMI < 30 groups (Appendix A, Table A2). No significant differences were observed in Fib-4 or NFS between the groups. In the BMI ≥ 30 group, ALT was higher in PiZZ individuals (*p* = 0.039). In the BMI < 30 group, the proportion of PiZZ individuals with pathologically elevated ALT was higher (*p* = 0.042) compared with PiMM individuals. No other differences in blood tests were statistically significant.

Both PiMM and PiZZ individuals with liver stiffness above 7.2 kPa were referred for further liver follow-up at the Department of Gastroenterology or to their attending physician at their local hospital.

In logistic regression analysis, AATD was associated with increased odds of liver stiffness compared with PiMM (OR 3.31, 95% CI 1.28–8.56). This association remained significant after adjustment for sex, BMI, and AUDIT score (Table 3). BMI was an independent risk factor for liver disease.

### 3.3. Results of Participating PiZZ Individuals with Liver Disease in Childhood

Of the 17 surviving individuals with liver disease in childhood, 8 (3 with severe and 5 mild liver disease) participated in this study. One with severe childhood liver disease had a TE value above 7.2 (9.2 kPa). In one individual with mild childhood disease, AST and ALT were pathologically elevated, while TE remained within the normal range. The remaining six participants showed normal liver assessments.

### 3.4. Liver Transplantation and Deaths During Ongoing Study

One PiZZ individual without a history of childhood liver disease underwent liver transplantation at the age of 48 years during the study. This individual developed cirrhosis followed by liver failure. Post-transplant examination showed a low platelet count (114 × 10^9^), while other laboratory values were within normal range, including a normal AAT level.

During the study period, six individuals died at the age of 47 (2), 48, 49, 50 and 51 years; however, none participated in the current study.

### 3.5. Liver Disease and Deaths in the Cohort Since Birth

During follow-up of the cohort since birth, 13 (10%) of the 129 PiZZ individuals had died. Overall mortality since birth was 10%. Liver disease was the immediate or underlying cause of death in 8 (62%) of these individuals. Of these, 3 deaths (23%) were attributed to alcohol-induced liver disease, corresponding to a liver-specific mortality of 6%. One additional individual underwent liver transplantation.

In total, 9 of 129 PiZZ individuals (7%) developed severe liver disease, including cirrhosis, liver failure, esophageal varices, and hepatorenal syndrome, resulting in either death or liver transplantation. Table 4 provides a summary of the number of deceased PiZZ individuals, their age at death, the presence of liver disease in childhood and adulthood, and the underlying and immediate causes of death.

## 4. Discussion

By the age of 50, individuals with severe AAT deficiency (PiZZ) identified through neonatal screening exhibited higher liver stiffness, measured by transient elastography (TE, FibroScan^®^), compared with an age-matched control group randomly selected from the general population. In contrast, liver fibrosis scores, as estimated by Fib-4 or NFS (Non-Alcoholic Fatty Liver Disease Fibrosis Score), did not differ significantly between the groups. Liver disease appeared to be the leading cause of mortality in this cohort by the age of 50, highlighting the potential importance of early detection and timely intervention to prevent disease progression.

To our knowledge, this is the first study to compare liver stiffness measured by TE between a cohort of PiZZ individuals and an age-matched control group randomly selected from the population registry. However, in our previous follow-up of this cohort at the age of 38 years, we observed significant differences in median liver stiffness between PiZZ and PiMM individuals using acoustic radiation force impulse (ARFI), an alternative measurement technique [13]. A possible explanation for higher liver stiffness in PiZZ individuals is the accumulation of a misfolded AAT protein (Z-type) in the periportal hepatocytes (zone I), leading to increased density within the liver mass, higher rate of hepatocyte injury and, as a consequence, elevated stiffness measurements [13,20].

Although the Fib-4 score is a well-established tool for excluding advanced liver fibrosis in individuals with Metabolic dysfunction-associated Steatotic Liver Disease (MASLD) [21], it did not differentiate between PiZZ and PiMM individuals in our study. One possible explanation for this discrepancy between Fib-4 and TE may be that the Fib-4 score was originally developed and validated in individuals with liver disease caused by hepatitis C infection (HCV) [12]. HCV is predominantly a zone III disease, in which most hepatocyte injury and necrosis occur in a centrilobular distribution [22,23,24]. Furthermore, MASLD, in which the Fib-4 score is widely used, also appears to predominantly involve zone III, particularly in the early stages of disease, albeit being a distinctly different entity than HCV-induced liver injury [25]. Compared with zone I, hepatocytes in zone III distribution have a lower concentration of ALT, whereas AST levels are similar in zone I and III [26].

Considering the formula for Fib-4 (*Fib-4* = (*Age* × *AST*)/(*Platelet count* × √*ALT*)), necroinflammation is required to elevate ALT in both HCV and MASLD. However, in AATD, misfolded Z-type AAT accumulates in zone I (periportal) hepatocytes, often without causing overt necrosis and therefore without marked ALT elevations in the early stages of the disease [27]. Since ALT is the denominator of the Fib-4 equation, even mildly elevated or normal ALT levels mathematically suppress the score, systematically underestimating fibrosis risk. This silent hepatocellular dysfunction may result in only modest ALT elevations, or even normal values, despite the presence of liver fibrosis.

Our results reflect this pattern. Despite higher TE values, suggesting a more advanced fibrosis in the PiZZ group, Fib-4 failed to capture this difference which demonstrates a biological and mathematical limitation of the Fib-4 in this context [28]. This highlights that relying solely on Fib-4 may result in under-detection of early fibrosis, supporting the use of complementary non-invasive tools such as elastography in clinical practice.

Although liver stiffness values on TE were higher—particularly among PiZZ individuals with BMI > 30, the Fib-4 score did not mirror these differences. The absence of correlation between elevated BMI and Fib-4, in contrast to the clear association between BMI and liver stiffness, indicates that metabolic stress in AATD may not be adequately captured by Fib-4. These findings underscore the need for fibrosis assessment tools that are more sensitive to the specific pathophysiological mechanisms underlying liver disease in PiZZ individuals.

Liver-related mortality at this age is generally uncommon in the general Swedish population [29], emphasizing the disproportionate contribution of hepatic complications, including cirrhosis, liver failure, esophageal varices, and hepatorenal syndrome, within this genetically defined cohort. In our cohort, liver disease accounted for 8 out of 13 deaths (6% of the total cohort) as either the immediate or underlying cause of death. Including one additional individual who underwent liver transplantation, 9 out of 129 (7%) developed severe liver disease resulting in death or transplantation. Given the limited cohort size, liver-related mortality numbers should be interpreted cautiously.

It should be noted that individuals who died from liver disease in adulthood did not participate in the most recent follow-up assessments, introducing potential survivor bias. Despite repeated invitations, some individuals declined or did not respond. In each invitation, participants were informed about the risks of both liver and lung disease associated with AAT deficiency and the importance of attending follow-up visits. Those attending the 50-year follow-up may therefore represent a more health-conscious or healthier subgroup, reflecting volunteer bias. Together, these factors may lead to an underestimation of the true burden of liver fibrosis in the original cohort.

Despite awareness of the risk of liver disease among the PiZZ individuals, their alcohol consumption remained high and did not differ significantly from that among the control group. Alcohol intake may therefore represent an additional behavioural factor that could interact with the underlying vulnerability to liver disease in this population.

These findings highlight the importance of addressing alcohol consumption among individuals with a genetic susceptibility to liver injury, such as patients with AATD. In our cohort, a considerable proportion of participants showed elevated PEth levels, and 3/13 (23%) of all deaths were attributed to alcohol-related liver disease, suggesting that alcohol consumption may act as an additional risk factor in susceptible individuals. While recent studies have suggested that moderate alcohol consumption may not markedly aggravate hepatic toxicity in PiMZ or PiZZ individuals [30], the impact of higher or sustained alcohol intake remains uncertain. Our observations therefore support the need for continued awareness and further investigation into the interaction between alcohol use and AATD-related liver disease.

In the regression analysis, the AUDIT score was not associated with liver stiffness, which may be explained by the limited number of individuals included in the analysis. It is also possible that the study subjects underestimated their alcohol consumption when reporting the AUDIT score. Further studies including a larger number of PiZZ individuals are needed to verify the association between alcohol consumption and liver disease.

## 5. Strength and Limitations

The cohort included in this study represents the only existing neonatal screening cohort of individuals with severe AATD followed into midlife, providing a unique opportunity to investigate the natural history of liver disease in this rare genetic disorder. To our knowledge, no other cohort of PiZZ individuals identified at birth has been followed for such an extended period. Between 1971 and 1974, 107,000 new-born children were screened in the Oregon State Public Health Laboratory, and 21 PiZZ individuals were identified [31]; the last follow-up of this group was published at the age of 15. In 1984–1985, 10,000 children were screened in Limburg, Belgium and six PiZZ individuals were identified [32]; no long-term follow-up has been published on these six PiZZ individuals.

The age-matched control group that was randomly selected from the population registry is an advantage, as age is a component of Fib-4 and NFSs and may otherwise cause confounding.

This study also has some limitations. As controls were selected at age 30 and followed longitudinally, there is a potential risk of accumulating comorbidities over time, which may affect comparability with PiZZ individuals at age 50. In addition, differences in follow-up participation between groups cannot be excluded. No re-weighting or adjustment for these factors was performed, which represents a limitation.

Another limitation is the relatively small number of participants, which reduces the statistical power, particularly in subgroup analyses. While 78% of PiZZ individuals responded to the questionnaires, participation in the clinical examination, including TE, was lower. Only 47 out of 122 PiZZ (39%) individuals underwent TE measurement, which may lead to bias. Despite several contact attempts and invitations to attend the study site, many participants reported being unable to attend due to work commitments, while others stated that they felt healthy and therefore did not consider a follow-up necessary, although they did complete the questionnaires.

A further limitation is the use of the M-probe for TE assessment, and the absence of the Controlled Attenuation Parameter (CAP) module in our FibroScan^®^ device; therefore, hepatic steatosis could not be quantified. Since steatosis may modestly influence liver stiffness measurements obtained by transient elastography, the absence of CAP data limits the ability to evaluate the potential contribution of fatty liver to the observed stiffness values. However, patients who required the X-probe or who showed elevated liver stiffness values on the FibroScan were referred to a gastroenterology clinic for reassessment with elastography using the X-probe.

Another limitation relates to the use of a liver stiffness cut-off of 7.2 kPa to define significant fibrosis. This threshold was derived from a relatively small cohort of individuals with AATD [10], and several different cut-off values have been proposed in the literature for assessing fibrosis in AATD-related liver disease [33,34,35,36,37]. Consequently, the optimal threshold for clinically relevant fibrosis in this population remains uncertain. Notably, a recent Delphi consensus document on the assessment of liver disease in AATD suggests a pragmatic cut-off of approximately 8 kPa for identifying individuals at increased risk of significant fibrosis [38]. The use of a slightly lower threshold in the present study may therefore influence the estimated prevalence of elevated liver stiffness, although it is unlikely to affect the overall comparative findings between PiZZ individuals and controls. Moreover, elastography-based fibrosis assessment entails etiology-specific cut-offs, which should be considered when interpreting liver stiffness values in different disease contexts [39].

## 6. Clinical Implication

An interesting finding is that, after the age of 8 years, the course of PiZZ AAT deficiency appears largely silent, with a latent period and no deaths. However, by the age of 40 (range 36–42 years), clinical events begin to occur, including liver disease and other complications—both alcohol- and non-alcohol-related. These results should be taken seriously.

Extrapolating from our longitudinal data, patient with the PiZZ genotype may be recommended to undergo regular hepatology assessment (every 1–2 years), with a particular focus on elastography starting at approximately age 40, along with proactive counselling regarding alcohol consumption and metabolic risk factors to reduce the risk of additive hepatotoxicity. These suggestions are, however, exploratory at best and should be interpreted in the context of the limited data presented in this paper.

In our cohort, one individual died by suicide, and two other individuals had excessive alcohol consumption. Although these observations are based on small numbers and no causal relationship can be inferred, they highlight the need for further studies on psychiatric health in individuals with severe AAT deficiency.

## 7. Conclusions

The current follow-up of this unique cohort shows that, in their early fifties, PiZZ individuals have early signs of liver fibrosis, measured by transient elastography. Our findings highlight a potential limitation of conventional serum-based fibrosis scores for detecting early liver fibrosis in genetically driven liver diseases characterized by periportal injury, such as Alpha-1-Antitrypsin deficiency. Liver disease and its complications represent the primary cause of death in PiZZ individuals by the age of 50.

These findings highlight the need for tailored screening and a structured, long-term follow-up to improve early detection and management of liver disease in PiZZ individuals.

## Figures and Tables

**Table 1:** Results of demographic data, alcohol consumption, blood samples, ultrasound of liver.

	PiZZ	PiMM	*p*-Value
**Demographic data**			
	n = 94	n = 124	
Men, n (%)	50 (53)	49 (40)	0.045
Age, years	49 (46–51)	49 (46–52)	NS
BMI	26.3 (20–44)	27.0 (16–40)	NS
Obesity, n (%)	14 (15)	29 (23)	NS
Metabolic syndrome, n (%)	5 (5)	17 (13)	0.042
Liver toxic medication, n (%)	12 (14)	28 (24)	NS
**Alcohol consumption**			
AUDIT score	3 (0–12)	3 (0–18)	NS
AUDIT score 8–15, n (%)	5 (6)	5 (4)	NS
PEth	n = 63	n = 96	
<35 ng/ml, n (%)	39 (62)	63 (66)	NS
35–200 ng/ml, n (%)	19 (30)	29 (30)	NS
>200 ng/ml, n (%)	5 (8)	4 (4)	NS
**Blood samples**			
	n = 68	n = 96	
AST (U/L)	25 (12–92)	24 (8–50)	NS
ALT (U/L)	28 (10–149)	23 (8–66)	0.007
GGT (U/L)	26 (10–128)	20 (7–126)	0.005
ALP (U/L)	66 (5–144)	63 (7–108)	NS
Bilirubin (µmol/L)	11 (2–29)	11 (2–29)	NS
Albumin (g/L)	44 (36–52)	44 (37–51)	NS
Platelets (×10^9^/L)	228 (126–397)	247 (132–501)	NS
AAT (mg/dL)	21 (14–32)	127 (96–205)	<0.001
**Ultrasound of liver**			
Ultrasound performed, n (%)	35 (37)	14 (11)	<0.01
Steatosis or fibrosis found in ultrasound, n (%)	4 (4)	2 (2)	NS

Data expressed as median (range) and frequency (percent); BMI: Body Mass Index; AUDIT: Alcohol Use Disorders Identification Test; PEth: Phosphatidyl-ethanol.

**Table 2:** Liver stiffness, fibrosis risk scores and ultrasound findings in participants undergoing transient elastography.

	PiZZ	PiMM	*p*-Value
**Transient elastography**			
	n = 47	n = 96	
Liver stiffness, kPa	5.9 (2.7–13.3)	4.5 (1.6–11.9)	<0.01
Liver stiffness over 7.2, n (%)	12 (26)	9 (9)	0.01
**Liver fibrosis risk score**			
	n = 44	n = 83	
Fib-4	0.95 (0.39–1.74)	0.93 (0.36–2.81)	NS
Fib-4 > 1.3, n (%)	10 (23)	16 (19)	NS
NFS	−2.6 (−4.2–−0.9)	−2.5 (−5.5–0.3)	NS
NFS > −1.455, n (%)	4 (10)	11 (13)	NS
**Ultrasound of liver**			
Ultrasound performed, n (%)	29 (60)	14 (14)	<0.01
Steatosis or fibrosis found in ultrasound, n (%)	3 (10)	2 (14)	NS

Data expressed as median (range) and frequency (percent); Fib-4: Fibrosis-4 index; NFS: Non-Alcoholic Fatty Liver Disease Fibrosis Score.

**Table 3:** Binary logistic regression analysis of predictors of liver stiffness > 7.2 kPa.

Variable	OR (95% CI)	*p*-Value
PiZZ vs. PiMM	3.39 (1.25–9.16)	0.016
BMI ≥ 30 vs. BMI < 30	3.10 (1.02–9.45)	0.047
Men vs. women	1.89 (0.67–5.37)	NS
AUDIT score ≥ 8 vs. AUDIT < 8	1.60 (0.29–8.94)	NS

BMI: Body Mass Index; AUDIT: Alcohol Use Disorders Identification Test.

**Table 4:** Summary of deaths among the cohort of 129 PiZZ individuals since birth, including sex, age at death, presence of liver disease, and causes of death.

Characteristic	N (%)
Total deaths	13 (10)
Sex	
Male	9 (69)
Female	4 (31)
Age at death	
<10 years	5 (38)
10–29 years	0
30–39 years	1 (8)
40–49 years	5 (38)
≥50 years	2 (15)
Infant individuals with liver disease	4 (31)
Adult individuals with liver disease in childhood	2 (15)
Liver disease as a contributing cause of death	8 (62)
Alcohol-induced	3 (23)
Cause of death	
Liver-related	7 (54)
Septicaemia	2 (15)
Respiratory failure	1 (8)
Accident or suicide	2 (15)
Unknown	1 (8)

## Data Availability

The data presented in this study are available on request from the corresponding author due to privacy or ethical reasons.

## Abbreviations

The following abbreviations are used in this manuscript:

AAT	alpha-1-antitrypsin
AATD	alpha-1-antitryspin deficiency
ALP	alkaline phosphatase
ALT	alanine transaminase
APRI	AST to platelet ratio index
AST	aspartate transaminase
ARFI	acoustic radiation force impulse
AUDIT	Alcohol Use Disorders Identification Test
BMI	body mass index
DRM-SUS	Department of Respiratory Medicine at Skåne University Hospital
ELF	enhanced liver fibrosis
ER	endoplasmatic reticulum
GGT	gamma-glytamyltransferase
HCC	hepatocellular cancer
LFT	liver function test
NFS	Non-Alcoholic Fatty Liver Disease Fibrosis Score
Peth	phosphatidylethanols
Pi	protease Inhibitor
SERPINA1	Serine Protease Inhibitor A1
TE	transient elastography
USS	ultrasound scan

## Appendix A

**Table A1:** Proportion of study participants (PiZZ and PiMM) with pathologically abnormal blood test results.

	PiZZ	PiMM	*p*-Value
	n = 68	n = 92	
AST, n (%)	7 (10)	5 (5)	NS
ALT, n (%)	5 (7)	0	0.007
GGT, n (%)	1 (2)	3 (3)	NS
ALP, n (%)	2 (3)	0	NS
Bilirubin, n (%)	1 (2)	2 (2)	NS
Albumin, n (%)	0	0	NS
Platelets, n (%)	2 (3)	1 (1)	NS

Data expressed as frequency (percent).

**Table A2:** Results of transient elastography, liver fibrosis score and blood tests stratified by BMI among participants who underwent transient elastography.

	BMI ≥ 30			BMI < 30		
	PiZZ	PiMM	*p*-Value	PiZZ	PiMM	*p*-Value
**Transient elastography**						
	n = 9	n = 21		n = 37	n = 75	
Liver stiffness, kPa	7.5 (6.3–9.2)	5.2 (4.2–6.4)	0.006	5.3 (4.3–6.7)	4.5 (3.7–5.3)	0.002
Liver stiffness over 7.2, n (%)	4 (44)	4 (19)	0.149	7 (19)	5 (7)	0.049
**Liver fibrosis risk score**						
	n = 8	n = 20		n = 37	n = 63	
Fib-4	0.88 (0.8–1.2)	0.88 (0.7–1.1)	0.636	0.99 (0.8–1.2)	0.98 (0.8–1.2)	0.991
Risk for liver fibrosis, n (%)	2 (25)	2 (10)	0.306	7 (20)	14 (22)	0.797
NFS	−2.0 (−2.5–−1.0)	−2.2 (−2.6–1.7)	0.709	−2.7 (−3.3–−2.4)	−2.5 (−3.1–−2.0)	0.365
Risk for advanced liver fibrosis, n (%)	3 (38)	4 (20)	0.334	1 (3)	7 (11)	0.167
**Blood samples**						
	n = 10	n = 21		n = 35	n = 72	
AST (U/L)	29 (24–34)	25 (18–31)	0.089	24 (22–29)	25 (19–29)	0.548
ALT (U/L)	31 (24–46)	24 (16–36)	0.039	26 (22–33)	23 (17–32)	0.060
GGT (U/L)	51 (29–57)	26 (18–37)	0.053	23 (19–29)	20 (13–26)	0.073
ALP (U/L)	75 (60–102)	72 (57–93)	0.244	66 (54–78)	66 (51–72)	0.196
Bilirubin (µmol/L)	8 (5–10)	9 (8–13)	0.546	11 (7–16)	11 (10–16)	0.631
Albumin (g/L)	44 (43–47)	44 (42–47)	0.724	45 (43–47)	44 (42–27)	0.370
Platelets (×10^9^/L)	285 (232–313)	265 (220–317)	0.983	227 (207–259)	244 (210–275)	0.709
**Pathologic blood samples**						
AST, n (%)	0	1 (5)	NS	2 (6)	4 (6)	0.987
ALT, n (%)	1 (10)	0	NS	2 (6)	0	0.042
GGT, n (%)	0	2 (10)	NS	0	1 (1)	0.481
ALP, n (%)	1 (10)	0	NS	0	0	NS
Bilirubin, n (%)	0	0	NS	1 (3)	2 (3)	0.991
Albumin, n (%)	0	0	NS	0	0	NS
Platelets, n (%)	0	0	NS	0	1 (1)	0.481

Data expressed as median (IQR) and frequency (percent). Fib-4: Fibrosis-4-index; NFS: Non-Alcoholic Fatty Liver Disease Fibrosis Score.

**Table A3:** Results of blood tests among participants who underwent transient elastography.

	PiZZ	PiMM	*p*-Value
	n = 47	n = 94	
AST (U/L)	25 (12–84)	25 (8–50)	NS
ALT (U/L)	28 (11–108)	23 (8–66)	0.005
GGT (U/L)	25 (10–72)	20 (7–126)	0.012
ALP (U/L)	66 (7–144)	66 (31–108)	NS
Bilirubin (µmol/L)	11 (2–29)	11 (2–29)	NS
Albumin (g/L)	44 (36–52)	44 (37–51)	NS
Platelets (×10^9^/L)	229 (174–397)	246 (132–501)	NS

Data expressed as median (range).

**Table A4:** Proportion of study participants (PiZZ and PiMM) with pathologically abnormal blood test results among participants who underwent transient elastography.

	PiZZ	PiMM	*p*-Value
	n = 47	n = 94	
AST, n (%)	3 (6)	5 (5)	NS
ALT, n (%)	3 (6)	0	0.013
GGT, n (%)	0	3 (3)	NS
ALP, n (%)	1 (2)	0	NS
Bilirubin, n (%)	1 (2)	2 (2)	NS
Albumin, n (%)	0	0	NS
Platelets, n (%)	0	1 (1)	NS

Data expressed as frequency (percent).
